# *redGEM*: Systematic reduction and analysis of genome-scale metabolic reconstructions for development of consistent core metabolic models

**DOI:** 10.1371/journal.pcbi.1005444

**Published:** 2017-07-20

**Authors:** Meric Ataman, Daniel F. Hernandez Gardiol, Georgios Fengos, Vassily Hatzimanikatis

**Affiliations:** Laboratory of Computational Systems Biotechnology, École Polytechnique Fédérale de Lausanne (EPFL), CH, Lausanne, Switzerland; Virginia Polytechnic Institute and State University, UNITED STATES

## Abstract

Genome-scale metabolic reconstructions have proven to be valuable resources in enhancing our understanding of metabolic networks as they encapsulate all known metabolic capabilities of the organisms from genes to proteins to their functions. However the complexity of these large metabolic networks often hinders their utility in various practical applications. Although reduced models are commonly used for modeling and in integrating experimental data, they are often inconsistent across different studies and laboratories due to different criteria and detail, which can compromise transferability of the findings and also integration of experimental data from different groups. In this study, we have developed a systematic semi-automatic approach to reduce genome-scale models into core models in a consistent and logical manner focusing on the central metabolism or subsystems of interest. The method minimizes the loss of information using an approach that combines graph-based search and optimization methods. The resulting core models are shown to be able to capture key properties of the genome-scale models and preserve consistency in terms of biomass and by-product yields, flux and concentration variability and gene essentiality. The development of these “consistently-reduced” models will help to clarify and facilitate integration of different experimental data to draw new understanding that can be directly extendable to genome-scale models.

## Author summary

Reduced models are used commonly to understand the metabolism of organisms and to integrate experimental data for many different studies such as physiology, fluxomics and metabolomics. Without consistent or clear criteria on how these reduced models are actually developed, it is difficult to ensure that they reflect the detailed knowledge that is kept in genome scale metabolic network models (GEMs). The redGEM algorithm presented here allows us to systematically develop consistently reduced metabolic models from their genome-scale counterparts. We applied redGEM for the construction of a core model for *E*. *coli* central carbon metabolism. We constructed the core model *irJO1366* based on the latest genome-scale *E*. *coli* metabolic reconstruction (*iJO1366*). *irJO1366* contains the central carbon pathways and other immediate pathways that must be connected to them for consistency with the *iJO1366*. *irJO1366* can be used to understand metabolism of the organism and also to provide guidance for metabolic engineering purposes. The algorithm is also designed to be modular so that heterologous reactions or pathways can be appended to the core model akin to a “plug-and-play”, synthetic biology approach. The algorithm is applicable to any compartmentalized or non-compartmentalized GEM.

## Introduction

Stoichiometric models have been used to study the physiology of organisms since 1980s [1–3], and with the accumulation of knowledge, and progressing techniques for genome annotation, these models have evolved into Genome Scale Metabolic Reconstructions (GEMs), which encapsulate all known biochemistry that takes place in the organisms by gene to protein to reaction (GPRs) associations [4]. Since the first Genome Scale models developed [5,6], the number of annotated genomes and the corresponding genome scale metabolic reconstruction has increased tremendously [7–9].

With increasing popularity of GEMs, different techniques to analyse these networks have been proposed [10,11]. Flux Balance Analysis (FBA), a constraint-based method (CBM) enables many forms of analysis based solely on knowledge of network stoichiometry and incorporation of various constraints, such as environmental, physicochemical constraints [12]. FBA has been further expanded by other methods such as Thermodynamics-based Flux Analysis (TFA) [13–16] and others [17,18] for the integration of available thermodynamics data with GEMs. TFA utilizes information about the properties of reaction thermodynamics and integrates them into FBA. Such properties now can be estimated by Group Contribution Method [19–21] and high-level Quantum Chemical Calculations[22]. Metabolic networks are valuable scaffolds that can also be used to integrate other types of data such as metabolic [23,24], regulatory and signalling [25–27], that can elucidate the actual state of the metabolic network *in vivo*. However, both FBA, TFA and other steady-state approaches cannot capture the dynamic response of metabolic networks, which requires integration of detailed enzyme kinetics and regulations [28]. Hatzimanikatis and colleagues have developed a framework that utilizes FBA, TFA and generates kinetic models without sacrificing stoichiometric, thermodynamic and physiological constraints [29–31]. Recently, another approach has been proposed to integrate kinetics into large-scale metabolic networks[32].

As the quality and the size of the models increase with better annotation, the complexity of the mathematical representations of the models also increases. Hatzimanikatis and colleagues [33] observed that majority of the studies and applications using metabolic models have still revolved around the central metabolism and around “reduced” models instead of genome-scale models, indicating that the full potential of GEMs remains largely untapped [34–38]. These reduced models have the advantage of having small sizes as they are built with a top-down manner, but they lack the quality of bottom-up built models since they have been reduced *ad hoc*, with different criteria and aims, which have not been consistently and explicitly justified [39–41]. An algorithmic approach called NetworkReducer [42] has been recently proposed following a top-down reduction procedure. The main purpose of this approach is to preserve selected so-called “protected” metabolites and reactions, while iteratively deleting the reactions that do not prevent the activity of the selected reactions. This algorithm has been further extended [43] to compute the minimum size of subnetworks that still preserve the selected reactions.

In this study, we have developed redGEM, a systematic model reduction framework for constructing core metabolic models from GEMs. Herewith, we propose an approach that focuses on selected metabolic subsystems and yet retains the linkages and knowledge captured in genome-scale reconstructions. redGEM follows a bottom-up approach that allows us to handle the complexity and to yield comprehensive insights in connecting the metabolic model to actual cellular physiology. redGEM can be tailored to generate minimal models with conserved functions. However, our approach is not strictly focused only on the reduction of the stoichiometry for the generation of highly condensed network, but aims also to preserve the constitutive characteristics of metabolic networks.

**Fig 1:**
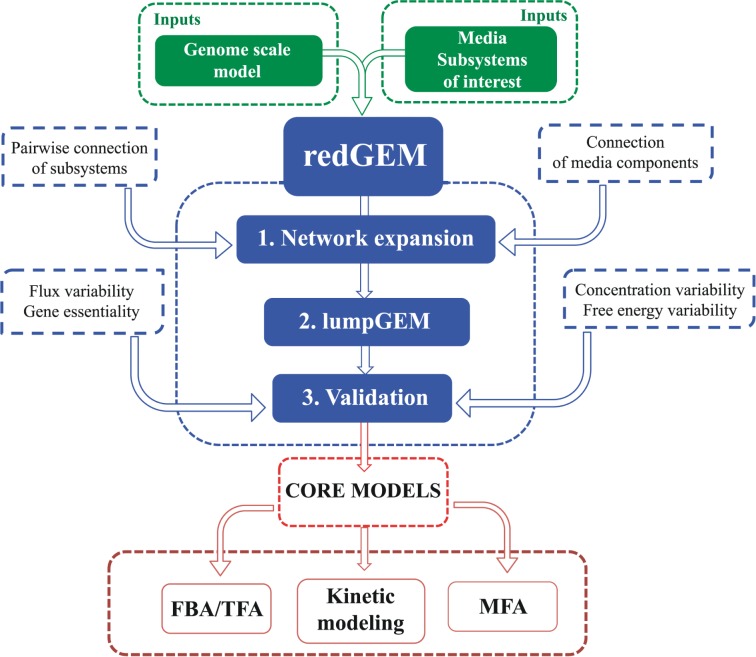
redGEM uses as inputs a GEM and the part of the metabolism of interest, along with the defined medium. With a 3 steps procedure that uses a set of methods, it generates core models for different purposes, such as FBA, TFA, kinetic modelling and metabolic flux analysis (MFA).

In redGEM, we use as inputs: (i) a GEM, (ii) metabolic subsystems that are of interest for a physiology under study; (iii) information about utilized substrates and medium components; and (iv) available physiological data (Fig 1). After a series of computational procedures, we generate a reduced model that is consistent with the original GEM in terms of flux profiles, essential genes and reactions, thermodynamically feasible ranges of metabolites and ranges of Gibbs free energy of reactions. We applied redGEM on the latest GEM of *E*. *coli iJO1366* [44] under both aerobic and anaerobic conditions with glucose and other possible carbon sources and generated a family of reduced *E*. *coli iJO1366* models.

## Results and discussion

We performed the redGEM algorithm on the latest GEM of *E*. *coli*, iJO1366 to generate a reduced model consistent with its parent GEM model. Firstly, we selected 6 central carbon metabolism subsystems (glycolysis, pentose phosphate pathway, citric acid cycle, glyoxylate cycle, pyruvate metabolism, and oxidative phosphorylation), as they are defined in original *E*. *coli* GEM. In addition, we have included all the reactions that use quinone/quinol pool metabolites (Ubiquinone/ubiquinol, menaquinone/menaquinol, 2- dimethyl menaquinone/2- dimethyl menaquinol for *E*. *coli*) in oxidative phosphorylation subsystem to capture the coupling between the core carbon metabolism and energy/redox metabolism. Some of those reactions had different subsystem definition in original GEM. These subsystems include a total of 185 reactions and 126 metabolites. We next redefined the content of each starting subsystem by performing an intra-expansion analysis to identify the *R*^*T*^ (See Material and Methods for definitions) reactions. We include a reaction in *R*^*T*^ when it only interconverts metabolites that are already included in one subsystem, and these reactions belong to a different subsystem in original GEM. This analysis established that there are many reactions in GEM whose reactants and products belong to a specific subsystem but are assigned to a different subsystem in the original GEM (Table 1). Some of the reactions defined in *R*^*T*^ are common between subsystems, since the subsystems share many metabolites, especially cofactor pairs such as ATP/ADP, NAD^+^/NADH etc.

**Table 1:** Statistics on starting subsystems with intra-expansion reactions, *R*^*T*^.

Subsystems	Metabolites	Reactions	Intra-Expansion Reactions
Citric Acid Cycle	24	10	6
Pentose Phosphate Pathway	21	12	2
Glycolysis/Gluconeogenesis	35	22	17
Pyruvate Metabolism	22	10	3
Glyoxylate Metabolism	13	4	3
Oxidative Phosphorylation	72	70	24
Media Composition	11	11	-

After the intra-expansion, the network expansion by directed graph search finds metabolites and reactions between subsystems in a pairwise manner for non-common metabolites (postulate 3 in Material and Methods) with respect to the degree of connection D. D is the distance between a subsystem pair and can be either equal to the inherent minimum distance between each pair, or imposed by the user for all subsystem pairs. Depending on the network topology, the inherent minimum distance can be equal to the input D imposed by the user (postulate 5 in Material and Methods). redGEM also performs pairwise connections between the metabolites of the same subsystem. The algorithm calculates *M*^*S*^, $M_{ij}^{D}$ and $M_{ii}^{D}$ (all pairs *i*, *j*), $R^{S_{i}}$, $R_{ij}^{D}$, $R_{ii}^{D}$ (all pairs *i*, *j*), which overall define the core network *CN*^*D*^ with respect to selected degree of connection parameter D (Table 2). The additional reactions for every degree of connection D are specific for the corresponding D (postulate 2 in Material and Methods). As a final step, redGEM performs an additional intra-expansion, and scans through every reaction in GEM to identify the reactions *R*^*T*^, which are not captured by $R^{S_{i}}$, $R_{ij}^{D}$, $R_{ii}^{D}$ (all pairs *i*, *j*) but include only *M*^*S*^, $M_{ij}^{D}$ and $M_{ii}^{D}$ (postulate 4 in Material and Methods). This procedure finalizes the steps that define the final core network for further analysis for redGEM. We performed redGEM on *E*. *coli* iJO1366 and we generated all core networks with degree of connection up to D = 6.

**Table 2:** The statistics of different Core Networks *CN*^*D*^.

Degree of Connection	# of Metabolites	# of Reactions
D = 0	126	185
D = 1	156	243
D = 2	197	286
D = 3	212	307
D = 4	227	324
D = 5	357	507
D = 6	461	653

The reported values for metabolites are compartmentalized, i.e. pyruvate cytoplasmic and pyruvate periplasmic are reported as different metabolites.

At D = 1, redGEM captured many connecting reactions that are part of many *ad hoc* built models, such as malic enzymes 1–2 between glycolysis and TCA cycle that connect L-malate to pyruvate, phosphoenolpyruvate carboxylase and phosphoenolpyruvate carboxykinase that connect oxaloacetate and phosphoenolpyruvate. Moreover, it captures many other reactions, such as 2 types of L-aspartate oxidases, which are using quinone/quinol cofactor pairs and labeled as electron transport chains reactions. There are two more L-aspartate oxidase reactions that are added to the D = 1 core network by redGEM (S1 Table). One uses O_2_/H_2_O_2_ and the other one is using fumarate/succinate as cofactor pairs. These reactions are captured by $R_{ii}^{D}$ and *R*^*T*^ simultaneously. Finally redGEM added 10 reactions whose reactants and products are only cofactors and small metabolites belonging to D = 1 core network in their stoichiometry, such as NAD^+^ kinase, NADP phosphatase, adenylate kinase, nucleoside-triphosphatase etc. as a part of *R*^*T*^. Along these reactions, the non-growth associated ATP maintenance (ATPM) reaction is explicitly included in the reduced model, and its corresponding minimum requirement of the GEM is preserved for further analysis in this study.

**Fig 2:**
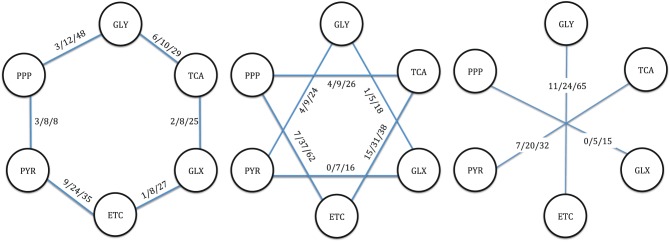
Pairwise connections between 6 intracellular starting subsystems. i/j/k represents the number of connecting reactions with respect to degree of connections D = 1, D = 2 and D = 3 respectively. The numbers are not cumulative, and represent the unique reactions for degree of connection. GLY: Glycolysis/ Gluconeogenesis, PPP: Pentose phosphate pathway, PYR: Pyruvate metabolism, ETC: Electron transport chain/Oxidative Phosphorylation, GLX: Glyoxylate metabolism, TCA: Tricarboxylic acid cycle.

When we analyze the pairwise connections between subsystems with respect to different connection parameter D, we observe that there is no D = 1 connection between certain pairs, such as pentose phosphate pathway (PPP) and glyoxylate metabolism (GLX) (Fig 2). However, zero connection between two subsystems by D = 1 does not necessarily mean that these subsystems are far from each other, as we observe that there are 5 and 15 reactions that are connecting PPP and GLX in 2 and 3 steps, respectively. As another extreme, tricarboxylic acid (TCA) cycle and electron transport chains (ETC) have 15 different reactions that connect each other with 1 reaction, demonstrating the strong connection between TCA cycle and redox metabolism.

Following the analysis for reactions, we identified the metabolites that connect the subsystems in a pairwise manner. There are no such intermediate metabolites between subsystems connected by D = 1, since this degree of connection only captures reactions between the unshared metabolites of a subsystem pair (Table 3). When the subsystems are connected pairwise with D = 2, there are 21 metabolites that become intermediates between all subsystem pairs. This number increases to 51 when degree of connection is increased to 3. By definition, a metabolite that connects a subsystem pair in 2 steps can also connect them in 3 steps through different reactions.

**Table 3:** Metabolites that connect subsystems and the number of pairwise connections they achieve with different degrees of connection parameter *D*. (According to postulate 1, see Material and Methods).

**Name of the connecting metabolite**	**D = 1**	**D = 2**	**D = 3**
D-tartrate	0	3	7
L-tartrate	0	3	7
Methylglyoxal	0	5	6
L-Malate	0	5	5
(R)-S-Lactoylglutathione	0	0	5
2-succinyl-5-enolpyruvyl-6-hydroxy-3-cyclohexene-1-carboxylate	0	4	4
Citrate	0	1	4
N6-(1,2-Dicarboxyethyl)-AMP	0	1	4
(S)-2-[5-Amino-1-(5-phospho-D-ribosyl)imidazole-4-carboxamido]succinate	0	1	3
CMP	0	1	3
D-Fructose	0	1	3
Glycerol	0	1	3
L-Aspartate	0	1	3
N(omega)-(L-Arginino)succinate	0	1	3
Oxaloacetate	0	1	3
5-O-(1-Carboxyvinyl)-3-phosphoshikimate	0	0	3
Chorismate	0	0	3
D-Glycerate 2-phosphate	0	0	3
Glyoxylate	0	0	3
Phosphoenolpyruvate	0	0	3
Formate	0	2	2
3-Phospho-D-glyceroyl phosphate	0	1	2
alpha,alpha-Trehalose 6-phosphate	0	1	2
D-Gluconate	0	1	2
alpha-D-Ribose 5-phosphate	0	0	2
D-Xylulose 5-phosphate	0	0	2
Hydroxypyruvate	0	0	2
L-Lactaldehyde	0	0	2
N-Carbamoyl-L-aspartate	0	0	2
D-Fructose 1,6-bisphosphate	0	1	1
D-Glucose	0	1	1
Dihydroxyacetone	0	1	1
2-Hydroxy-3-oxopropanoate	0	0	1
Adenosine 3,5-bisphosphate	0	0	1
AMP	0	0	1
Citrate	0	0	1
D-Erythrose 4-phosphate	0	0	1
D-Gluconate	0	0	1
D-Lactate	0	0	1
D-Ribulose 5-phosphate	0	0	1
Glyceraldehyde 3-phosphate	0	0	1
Glycerol 3-phosphate	0	0	1
Maltoheptaose	0	0	1
Maltohexaose	0	0	1
Maltopentaose	0	0	1
Pyruvate	0	0	1
R-Glycerate	0	0	1
Sedoheptulose 1,7-bisphosphate	0	0	1
Sedoheptulose 7-phosphate	0	0	1
Succinate	0	0	1
Trehalose	0	0	1

There are metabolites, such as pyruvate and succinate, that already participate in D = 0 reactions (in the initial starting subsystems), and they appear later to connect at least one subsystem pair with D = 3 connection. This indicates that there is no path in GEM with length less than 3 that can connect any starting subsystem pair using these intermediates, excluding the reactions that already belong to this subsystem pair.

Methylglyoxal is known to be a hub metabolite, since it can connect dihydroxyacetone phosphate to lactate in 2 reactions. Lactate is a metabolite that belongs to different starting D = 0 subsystems such as oxidative phosphorylation and pyruvate metabolism. Moreover, it can be converted to pyruvate by lactate dehydrogenase, and pyruvate is already known as a hub metabolite that can connect different subsystems. As another example, L and D tartrate connect pentose phosphate pathway and citrate cycle in 3 steps through the following path: With an antiporter, cytosolic succinate transports L and D forms of tartrate to cytosol. Then, L and D-tartrate dehydratase enzymes convert these two forms of tartrate to oxaloacetate and water. Following this biotransformation, oxaloacetate can be converted to pyruvate by many enzymes. As we observed in methylglyoxal case, pyruvate is part of many different starting D = 0 subsystems (glycolysis/gluconeogenesis, oxidative phosphorylation, citrate cycle, pyruvate metabolism and extracellular subsystem), and L and D tartrate appear as intermediates that connect 7 pairs of subsystems in D = 3.

Another layer of information that we can extract through this analysis is the subsystems that connect the selected starting subsystems, thus demonstrating the proximity of these subsystems to the defined starting core carbon ones. By starting from 7 subsystems (including extracellular metabolites as extracellular subsystem), the network expansion procedure results in capturing reactions as core from 32 different subsystems for D = 6 (Table 4). In GEM, there are 37 subsystems, which signifies that only 6 steps expansion captures reactions from ~90% of all subsystems defined in GEM, thus showing the tight connections between metabolites/subsystems in the network. For 2 subsystems defined in GEM, anaplerotic reactions and methylglyoxal metabolism, more than half of the all reactions within these subsystems are captured by network expansion procedure with connection parameter D being up to 3 (Table 5). An important observation is that components of the same subsystems can be parts of the connection of more than 1 subsystem pairs, since different subsystems can share the same metabolites.

**Table 4:** The subsystems that can be reached from starting subsystems in 6 steps.

SUBSYSTEMS REPRESENTED IN D = 6 CORE NETWORK	
Alanine and Aspartate Metabolism	Histidine Metabolism
Alternate Carbon Metabolism	Inorganic Ion Transport and Metabolism
Anaplerotic Reactions	Lipopolysaccharide Biosynthesis / Recycling
Arginine and Proline Metabolism	Methionine Metabolism
Cell Envelope Biosynthesis	Methylglyoxal Metabolism
Tricarboxylic acid cycle	Murein Recycling
Cofactor and Prosthetic Group Biosynthesis	Nucleotide Salvage Pathway
Cysteine Metabolism	Pentose Phosphate Pathway
ETC Rxns–Oxidative Phosphorylation	Purine and Pyrimidine Biosynthesis
Exchange	Pyruvate Metabolism
Folate Metabolism	Threonine and Lysine Metabolism
Glutamate Metabolism	Transport, Inner Membrane
Glycerophospholipid Metabolism	Transport, Outer Membrane Porin
Glycine and Serine Metabolism	Tyrosine, Tryptophan, and Phenylalanine Metabolism
Glycolysis/Gluconeogenesis	Unassigned–No subsystem association
Glyoxylate Metabolism	Valine, Leucine, and Isoleucine Metabolism

**Table 5:** The subsystems that are connecting 6 starting subsystems.

	TCA PPP	TCA GLY	TCA PYR	TCA GLX	TCA ETC	PPP GLY	PPP PYR	PPP GLX	PPP ETC	GLY PYR	GLY GLX	GLY ETC	PYR GLX	PYR ETC	GLX ETC	Total
Alanine and Aspartate Metabolism	0/0/1	0/0/2	0/2/2	0/0/0	2/2/2	0/0/0	0/0/0	0/0/0	0/1/2	0/0/0	0/0/0	0/1/2	0/0/0	1/2/2	0/0/1	2/2/2 (%22/%22/%22)
Alternate Carbon Metabolism	0/0/3	0/0/3	2/4/3	0/0/1	1/3/3	2/7/21	0/0/0	0/0/0	1/3/10	0/0/1	0/1/4	2/5/13	0/0/0	2/4/3	0/1/4	6/17/29 (%3/%9/%15)
Anaplerotic Reactions	2/2/4	4/4/6	2/2/4	2/2/6	4/4/4	0/1/3	0/2/0	0/3/3	2/3/3	2/4/4	0/3/4	4/5/5	0/3/5	2/3/3	1/3/5	6/6/6 (%75/%75/%75)
Arginine and Proline Metabolism	0/0/0	0/0/0	0/0/2	0/0/0	0/2/2	0/0/0	0/0/0	0/0/0	0/0/0	0/0/0	0/0/0	0/0/0	0/0/0	0/0/2	0/0/0	0/2/2 (%0/%5/%5)
Cofactor and Prosthetic Group Biosynthesis	0/2/4	0/2/4	0/3/4	0/0/2	2/5/5	0/0/1	1/1/1	0/0/0	0/1/4	0/0/2	0/0/0	0/0/4	0/0/0	0/2/2	0/0/2	3/6/8 (%1/%3/%4)
Cysteine Metabolism	0/0/0	0/0/0	0/0/0	0/0/0	0/0/0	0/0/0	0/0/0	0/0/0	0/0/1	0/0/0	0/0/0	0/0/0	0/0/0	0/0/0	0/0/0	0/0/1 (%0/%0/%8)
Glycerophospholipid Metabolism	0/0/0	0/0/0	0/0/0	0/0/0	0/0/0	0/0/7	0/0/0	0/0/0	0/7/7	0/0/0	0/0/0	0/0/7	0/0/0	0/0/0	0/0/0	0/7/7 (%0/%3/%3)
Glycine and Serine Metabolism	0/0/0	0/0/0	0/0/0	0/0/1	0/0/0	0/0/0	0/0/0	0/0/0	0/0/0	0/0/0	1/1/1	0/0/1	0/0/1	0/0/0	0/0/1	1/1/1 (%7/%7/%7)
Methylglyoxal Metabolism	0/0/0	0/0/0	0/0/0	0/0/0	0/0/0	0/0/2	0/2/4	0/0/0	0/2/5	0/2/4	0/0/0	0/2/5	0/0/0	0/2/4	0/0/0	0/2/5 (%0/%22/%56)
Nucleotide Salvage Pathway	0/0/0	0/0/0	0/0/0	0/0/0	0/0/0	0/0/1	0/0/0	0/0/0	0/1/2	0/0/0	0/0/0	0/0/1	0/0/0	0/0/0	0/0/0	0/1/2 (%0/%1/%1)
Purine and Pyrimidine Biosynthesis	0/0/1	0/0/1	0/0/5	0/0/1	1/5/7	0/0/0	0/0/0	0/0/0	0/0/3	0/0/0	0/0/0	0/0/1	0/0/0	0/0/7	0/0/1	1/5/7 (%4/%22/%30)
Transport, Inner Membrane	0/1/6	0/1/6	0/5/6	0/1/3	4/6/10	1/3/4	0/0/0	0/0/0	1/6/11	0/0/0	0/0/0	3/5/9	0/0/0	2/6/7	0/1/3	9/11/14 (%3/%3/%4)
Tyrosine, Tryptophan, and Phenylalanine Metabolism	0/0/0	0/0/0	0/0/0	0/0/0	0/0/0	0/0/3	0/0/0	0/0/0	0/0/0	0/0/3	0/0/0	0/0/3	0/0/0	0/0/0	0/0/0	0/0/3 (%0/%0/%13)

i/j/k represents the number of reactions that belong to the new subsystem, which connect a starting subsystem pair with respect to degree of connection parameter D = 1, D = 2 and D = 3, respectively. Percentage refers to the percentage of the total number of reactions connecting all 6 pairs over the total number of reactions in GEM labeled with the corresponding subsystem.

### Generation of lumped reactions for biomass building blocks from core carbon network

**Fig 3:**
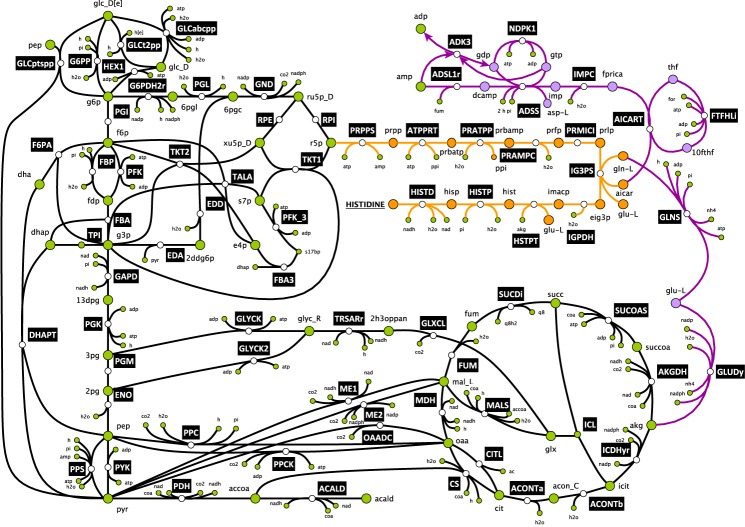
The synthesis of histidine from core carbon network. Histidine synthesis starts from ribose-5-phosphate (R5P) from Pentose Phosphate Pathway, and consists of 10 reaction steps. Not all reactions of core network are shown. Orange reactions form the linear pathways for histidine as defined in databases. Metabolites colored with green are *core* metabolites, whereas orange and purple metabolites are *non-core*. The *non-core* metabolites along the linear pathway for histidine synthesis are balanced by the purple reactions.

The wild-type biomass reaction of the iJO1366 model contains 102 biomass building blocks (BBBs). The size and the complexity of the composition makes it necessary to develop techniques to keep the information stored in GEM for the biosynthesis, but yet reduce the size of the network significantly. Methods, such as graph-search algorithms can be used for identification of biosynthetic routes between two metabolites in metabolic networks [45,46]. However, these graph theory based approaches cannot be used for our purposes due to two main issues and limitations: *i) they do not make use nor obey mass conservation; hence the pathways they generate are not guaranteed to be able to carry flux in metabolic network or to be elementally balanced*, *ii) and they cannot manage pathways that are not linear*, *such as branched pathways*. To overcome these limitations, we used lumpGEM [47], an in-built tool, which identifies subnetworks that can produce biomass building blocks starting from precursor metabolites. These precursors are provided by redGEM through the systematically generated core network based on degree of connection parameter, D. Each subnetwork is then transformed into a lumped reaction and inserted in the reduced model. lumpGEM forces mass conservation constraints during optimization to identify subnetworks, thus preventing the generation of lumped reactions, which cannot carry flux in the metabolic networks. As an example, for D = 1, by minimizing the number of *non-core* reactions In GEM, lumpGEM generated a 17 reactions subnetwork to synthesize histidine from *core* carbon metabolites (Fig 3). Histidine is synthesized from ribose-5-phosphate, a precursor from pentose phosphate pathway. The linear pathway from this *core* metabolite to histidine is composed of 10 steps. However, due to the mass balance constraint, two metabolites, 1-(5-Phosphoribosyl)-5-amino-4-imidazolecarboxamide and L-Glutamine cannot be balanced in a network that is composed of *core* reactions and the linear pathway from ribose-5-phophate to histidine. These metabolites are balanced in the network by other *non-core* reactions. Hence, the generated sets of reactions are not linear routes from precursor metabolites to biomass building blocks, but *branched*, *balanced subnetworks* (for formulation of lumpGEM, see Material and Methods).

Using lumpGEM, we replicated all the biosynthetic pathways in databases such as EcoCyc [48], either as a part of subnetworks or the exact pathway. In addition, we identified subnetworks that can qualify as alternative biosynthetic pathways. *E*. *coli* is well-known to be robust against deletions by having many duplicate genes and alternate pathways[49]. Some of these routes may not be active due to energetics or regulatory constraints but using lumpGEM we can map these possible alternate pathways completely and also derive different biosynthetic lumped reactions. The introduction of such lumped biosynthetic reactions simplifies the core models considerably and allows the use of these models in important computational analysis methods such as dynamic FBA [50] extreme pathway analysis [51,52] and elementary flux modes [53,54], as well as for TFA formulations and kinetic modeling.

For D = 1 core network, lumpGEM generated 1216 subnetworks and 254 unique lumped reactions for 79 biomass building blocks in total for aerobic and anaerobic case. The remaining BBBs of the total 102 can be produced within the D = 1 core network. For some biomass building blocks, it is possible that all the alternatives for *S*_*min*_ (the minimal subnetwork size) subnetworks generated under aerobic conditions are using molecular oxygen, thus cannot carry flux under anaerobic conditions. This necessitates the generation of lumped reactions without any oxygen in the media. For *S*_*min*_, lumpGEM generated only 4 new lumped reactions for anaerobic case, for 3 metabolites, namely, heme O, lipoate (protein bound) and protoheme. All the other lumped reactions generated for anaerobic case are a subset of the 250 lumped reactions (S2 Table) for aerobic conditions. In the subsequent studies, we used all lumped reactions in order to allow for studies under different oxygen limitations without changing the model structure. The core model can be found in the supplementary material (S1 File).

### Validation

#### Maximum biomass under different carbon sources

One of the most important criteria for the reduced GEM (rGEM) validation is the maximum biomass production. We performed biomass maximization with FBA and TFA. With all 254 lumped reactions, maximum specific growth rate of the rGEM is the same as GEM’s μ_*max*_, 0.99 hr^-1^ with 10 mmol/gDWhr glucose uptake rate under aerobic conditions both with FBA and TFA. The anaerobic specific growth rate of GEM with the same carbon source for FBA is ~0.67/hr and with thermodynamic constraints (TFA) it drops to 0.27/hr. rGEM grows with 0.27/hr specific growth rate both with FBA and TFA. When we analyzed the discrepancy between the FBA and TFA growth rates for GEM, we saw that the difference is emerging from reactions that use molecular oxygen in GEM. These oxygen-using reactions do not belong to oxidative phosphorylation or ETC reactions, and are not a part of rGEM network. Moreover, the standard Gibbs free energy of those reactions range from 19kcal/mol to 294 kcal/mol in the oxygen producing direction[21] and are thermodynamically infeasible, except for 5 reactions which are mainly degradation of hydrogen peroxide and superoxide anion. These 5 reactions have no effect on growth rate.

To incorporate experimental fluxomics data to validate the model, we included 13C-MFA data from Haverkorn et al. [55]. In this study, the authors estimated the fluxes for core carbon metabolism, the uptake and secretion of the cell, and the corresponding specific growth. By incorporating 22 fluxes, along with the specific uptake rate of glucose, both the GEM and the reduced model predicted the specific growth rate as 0.65/hr, which is very close to the observed value as 0.61/hr. This overestimation from the GEM was expected, mainly because the 13C-MFA data is not enough to constrain the model to the experimentally observed physiology. However our objective in this study is to preserve the consistency between rGEM and GEM, and this consistency is still kept with the additional experimental data.

The core networks generated by redGEM are the same for different possible carbon sources, since they are incorporated in the core network as extracellular subsystem. Thus, the only difference that can emerge for the reduced models growing on different substrates will be the generated lumped reactions. Before rerunning the lumpGEM algorithm under different environmental conditions, we tested specific growth rate of the reduced model generated under glucose for different carbon sources (Table 6). The theoretical optimum yield is the same for different possible carbon sources between rGEM and GEM except formate. GEM can grow on formate very slowly (0.034/hr), whereas rGEM does not grow on formate at all, with the lumped reactions generated under glucose. Thus, to generate a reduced model growing on formate, the lumping procedure should be repeated.

**Table 6:** The specific growth rates (/hr) with 10 mmol/gDWhr uptake for each carbon source.

Carbon Source	GEM	rGEM
**Glucose**	0.998	0.994
**Succinate**	0.506	0.505
**Acetate**	0.260	0.259
**Ethanol**	0.434	0.432
**Glycerol**	0.577	0.576
**Lactate**	0.441	0.439
**Alpha-ketoglutarate**	0.631	0.628
**Formate**	0.034	0.000
**Pyruvate**	0.372	0.371
**Malate**	0.496	0.494

To compare the subnetworks and lumped reactions under different carbon sources and environmental conditions, we generated a reduced model of *E*. *coli* iJO1366 growing under glycerol anaerobically. The growth rates of rGEM and GEM under these conditions with TFA are the same, 0.113/hr. The number of generated *S*_*min*_ subnetworks under glycerol is 910, compared to 1212 generated under aerobically grown *E*. *coli* with glucose as sole carbon source. There are 237 unique lumped reactions for growth under glycerol, whereas there are 250 unique lumped reactions for glucose case. Among these 237 lumped reactions for glycerol, there are only 8 different lumped reactions compared to glucose case for heme O, protoheme and lipoate (protein bound).

#### Gene essentiality comparison between rGEM and GEM

One of the most common analyses for genome-scale models is *in silico* gene deletion (knockout) experiments to *i) identify essential and nonessential genes*, *ii) study the gene deletion impact on the organism physiology*, *iii) develop strategies for metabolic engineering* [56]. Consistency of gene knockouts between rGEM and GEM is another important corroboration for the reliability of the reduction procedure. *irJO1366*, generated with D = 1, shares 307 genes with GEM, and among these 307 genes, 25 are essential. 22 of those genes are also essential in GEM. 2 out of 3 conflicting genes do not have an effect on the maximum theoretical yield of *E*. *coli* under aerobic, minimal glucose medium in the GEM. The first case is the gene transcribing thioredoxin reductase enzyme, which interconverts NADPH to NADP by using oxidized thioredoxin and reduced thioredoxin as cofactor pairs. This reaction is not essential in GEM, however it is essential in rGEM, since the cofactor pair oxidized thioredoxin and reduced thioredoxin participate in lumped reactions, and due to flux coupling, the reaction that thioredoxin reductase catalyzes becomes indispensible. We searched for alternative lumped reactions so that this gene will not be essential in rGEM. However, lumped reactions constructed from *S*_*min*_ do not make this gene non-essential. The second discrepancy of the responses to gene deletion between rGEM and GEM is the gene transcribing Glutamate dehydrogenease, which shows a different behaviour compared to thioredoxin reductase enzyme. The reaction it catalyzes is the only reaction that synthesizes glutamate in the rGEM, and knocking out this enzyme automatically results in no specific growth rate. Deleting this enzyme in GEM results in a growth rate drop of 3.3%, and alternative synthesis pathways for glutamate in GEM abolishes the essentiality. The deletion of adenylate kinase (*adk*) is the third discrepancy between rGEM and GEM. Knocking out this gene does not result in any drop in growth rate for GEM, however it prevents the cellular growth in rGEM. The reason for this discrepancy is similar to the case of thioredoxin reductase, i.e. the loss of alternative reactions/pathways that can complement this deletion. Although these reactions/pathways can be a part of the subnetworks, the corresponding lumped reactions cannot add such flexibility in the rGEM network. Interestingly *adk* is reported as essential in literature[57], thus showing that the alternative pathways that compensate for the loss *adk* gene in GEM are either not active or not catalytically efficient/favorable.

#### Flux and thermodynamic-based variability analysis–comparison between rGEM and GEM

**Fig 4:**
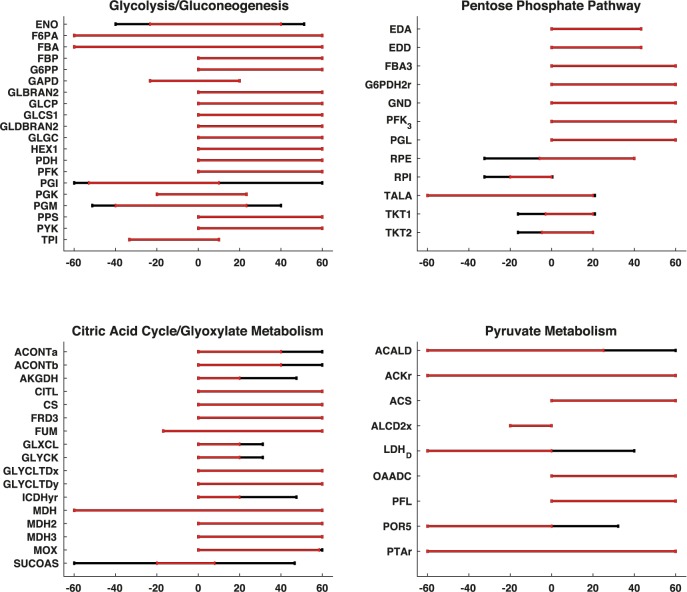
Flux variability of reactions in starting subsystems in D = 1 model compared to corresponding reactions in GEM. The red lines represents FVA for redGEM, black lines represents FVA for GEM. There cannot be any reaction in rGEM that has a wider range than corresponding GEM reaction. Thus, for the reactions that do not have the black line have the same range for rGEM and GEM. Maximum flux bounds are between -60 to 60 mmol/gDWhr, since the uptake of glucose is fixed to 10 mmol/gDWhr, and the maximum allowable flux in the network cannot exceed 10 mmol/gDWhr times 6, which is the number of carbon in glucose.

To further validate the model, we compared the physiologically allowable flux ranges with flux variability analysis (FVA), allowable concentration ranges for metabolites and Gibbs free energy of reactions by performing Thermodynamics-based Variability Analysis (TVA) for the reactions and metabolites that are common between rGEM and GEM. Comparisons for the allowable flux ranges revealed that most of the common intracellular reactions between rGEM and GEM have consistent flux ranges, however, there are some reactions in the rGEM with reduced flux variability as compared to GEM counterparts (Fig 4). The variability of the reactions in the subsystems glycolysis/gluconeogenesis, pentose phosphate pathway, and citric acid cycle of rGEM are close to variability in GEM, due to the nature of the construction of rGEM, which is built by expansion of these subsystems and this expansion includes all the close links that allow the flux variability of the reactions in these subsystems. Reactions that belong to pyruvate metabolism and electron transport chains (ETC) show a higher variability in GEM compared to rGEM, due to the alternative reactions that use metabolites from these subsystems and are not a part of the reactions in the network expansion. Another main difference between rGEM and GEM emerges from the reaction directionalities, since rGEM is more constrained, some reactions, such as LDH (Lactate Dehydrogenase) become unidirectional. Moreover, as we discussed in the case of essentiality studies, the integration of reactions into lumped reactions reduces the flexibility of the flow in the network.

We next performed a Concentration Variability Analysis (CVA) (within TVA) on common metabolites between rGEM and GEM (S1 Fig). Almost all metabolites have the same allowable ranges with a few exceptions. Succinly-CoA and D-Ribulose 5-phosphate are two such cases, where rGEM bounds are wider than GEM bounds. Succinly-CoA participates in reaction tetrahydrodipicolinate succinylase with CoA as cofactor pair in GEM, but not in rGEM. Succinly-CoA concentration is tightly constraint due to bioenergetics to synthesize N-Succinyl-2-L-amino-6-oxoheptanedioate, which is an intermediate in L-lysine biosynthesis. The lumped reactions for L-lysine subnetworks do not include this metabolite in the overall stoichiometry, since it is an intermediate and hence, Succinly-CoA concentration is not constrained in the rGEM. Showing the same behaviour, D-Ribulose 5-phosphate concentration is constrained in arabinose-5-phosphate isomerase reaction in GEM, which is in Lipopolysaccharide biosynthesis pathway and this reaction is involved in a lumped reaction in rGEM.

## Conclusion

Reduced models have been used to understand and investigate cellular physiology for many years. Before the emergence of genome scale models (GEMs), different groups with different aims built reduced models for their studies with a top-down approach. Conversely, GEMs provide the platform to understand all the metabolic capabilities of organisms, since GEMs encapsulate all the known biochemisty that occurs in cells. However the complexity of GEMs make their use impractical for different applications, such as kinetic modeling or elementary flux modes (EFMs). The need to focus on certain parts of these networks without sacrificing detailed stoichiometric information stored in GEMs makes it crucial to develop representative reduced models that can mimic the GEM characteristics. Within this scope, we developed redGEM, an algorithm that uses as inputs genome-scale metabolic model and defined metabolic subsystems, and it derives a set of reduced core metabolic models. These family of core models include all the fluxes across the subsystems of interest that are identified through network expansion, thus capturing the detailed stocihiometric information stored in their bottom-up built parent GEM model. Following the identification of the core, redGEM uses lumpGEM, an algortihm that captures the minimal sized subnetworks that are capable of producing target compounds from a set of defined core metabolites. lumpGEM expands these core networks to the biomass building blocks through elementally balanced lumped reactions. Moreover, redGEM employs lumpGEM to include alternative lumped reactions for the synthesis of biomass building blocks, thus accounting for alternative sytnhesis routes that can be active under different physiological conditions.

redGEM builds reduced models rGEMs that are consistent with their parent GEM model in terms of flux and concentration variability and essential genes/reactions. These reduced models can be used in many different areas, such as kinetic modeling, MFA studies, Elementary Flux Modes (EFM) and FBA/TFA. redGEM algorithm is applicable on any compartmentalized or non-compartmentalized genome scale model, since its procedure does not depend on any specific organism. As a demonstration, we have applied the redGEM algorithm on different organisms, namely *P*. *putida*, *S*. *cerevisiae*, Chinese Hamster Ovary cell (CHO) and human metabolism. For instance, redGEM algorithm has generated core networks of sizes between 168 metabolites/164 reactions to 360 metabolites/414 reactions for iMM904 [58] GEM reconstucted for *S*. *cerevsiae* with degree of connection parameter D varied from 1 to 6. The generated reduced model *irMM904* with D = 1 has the same biomass yield with the parent model GEM as 0.29/hr under 10 mmol/gDWhr glucose uptake. Similar to *E*. *coli* case, flux and concentration variability, and gene essentiality characteristics of the rGEM are in agreement with the GEM counterparts (Ataman et al., manuscript in preparation). Moreover, reduced models are promising platforms for the comparison of central carbon (or any other) metabolism of different species. This approach can help us to better investigate the metabolic capabilities and limitations of organisms and to identify the sources of physiological differences across different species.

## Materials and methods

We applied redGEM algorithm on the latest genome scale model of *E*. *coli* iJO1366 [44], which is composed of 2251 enzymatic reactions (including transporters), 1136 unique metabolites across cytoplasm, periplasm and extracellular media. We used glucose as the sole carbon source and constrained the model for aerobic conditions.

### Preliminary definitions

In redGEM, we introduce and use the following definitions:

*S*_*i*_: Core subsystem *i* that is selected/defined by the user.
$M^{S_{i}}$: Metabolites that belong to subsystems *S*_*i*_.
$R^{S_{i}}$: Reactions that belong to subsystems *S*_*i*_.
*Degree of connection D*: The path length between two subsystems. It corresponds to the number of reactions that link subsystems *S*_*i*_ and *S*_*j*_.
$R_{ij}^{D}$: The reactions in all paths of length *D* between the subsystems *S*_*i*_ and *S*_*j*_; these reactions do not belong to either $R^{S_{i}}$ or $R^{S_{j}}$.
$M_{ij}^{D}$: The metabolites that are intermediates in all paths of length *D* between the subsystems *S*_*i*_ and *S*_*j*_; these metabolites do not belong to either $M^{S_{i}}$ or $M^{S_{j}}$.
Postulate 1: Reactions that belong to $R_{ij}^{D}$ and metabolites that belong to $M_{ij}^{D}$ can belong to any of the subsystems *S*_*m*_ with *m* ≠ *i and m* ≠ *j*.
Postulate 2: Some of the reactions in $R_{ij}^{D+n}$ can belong to $R_{ij}^{D}$. Reactions in $R_{ij}^{D=n}$ that do not belong in any other $R_{ij}^{D=1,2,..}\;(where\;D\neq n)$ are called *unique reactions* for the degree of connection *D*.
Postulate 3: $R_{ij}^{D}$ and $M_{ij}^{D}$ captures the connections between the non-common metabolites of *S*_*i*_ and *S*_*j*_, however it cannot capture the intra-connections between the metabolites of the same subsystem or the metabolites that are shared between *S*_*i*_ and *S*_*j*_.
$R_{ii}^{D}$: The reactions in all paths of length *D* that intra-connects the metabolites of the subsystem *S*_*i*_.
$M_{ii}^{D}$: The intermediate metabolites in all paths of length *D* that intra-connects the metabolites of the subsystem *S*_*i*_.
*R*^*T*^: Reactions where only $M^{S_{i}}$, $M_{ij}^{D}$ and $M_{ii}^{D}$, participate and do not belong to $R^{S_{i}}$, $R_{ij}^{D}$ and $R_{ii}^{D}$.
Postulate 4: *R*^*T*^ is composed of reactions that only cofactor pairs, small metabolites and inorganics participate. All the other reactions that include other core metabolites (along with cofactor pairs, small metabolites and inorganics) will be a part of $R^{S_{i}}$, $R_{ij}^{D}$ or $R_{ii}^{D}$.
Core Network, *CN*^*D*^: The core network for redGEM that is composed of metabolites $M^{S_{i}}$, $M_{ij}^{D}$ and $M_{ii}^{D}$, and of reactions $R^{S_{i}}$, $R_{ij}^{D}$, $R_{ii}^{D}$ and *R*^*T*^.
rGEM: Consistently reduced model from its parent GEM.

We can also generate the core network from the chosen subsystems using the minimum distance between the chosen subsystems and report the connecting reactions and metabolites. In this case, the degree of connection *D* is the minimum distance between *S*_*i*_ and *S*_*j*_.

*L*_*min*,*ij*_: The length of the shortest path between the subsystems *S*_*i*_ and *S*_*j*_.
$R_{i,j}^{L_{min+n,ij}}$: The reactions that connect the subsystems *S*_*i*_ and *S*_*j*_ with a path of length *L*_*min*+*n*,*ij*_ in where *n* is a user defined parameter.
$M_{\;ij}^{L_{min+n,ij}}$: The metabolites that do not belong to either *S*_*i*_ or *S*_*j*_ and are intermediates of the path of length *L*_*min*+*n*,*ij*_ in between these two subsystems.
Postulate 5: If *L*_*min*,*ij*_ = 1 then $R_{ij}^{L_{min,ij}},\;M_{ij}^{L_{min,ij}}$ becomes $R_{ij}^{1},\;M_{ij}^{1}$, this also results in $R_{ij}^{K},M_{ij}^{K}=R_{ij}^{L_{min+(K-1),ij}},M_{ij}^{L_{min+(K-1),ij}}$.

### redGEM parameters

redGEM uses the following inputs and parameters:

1. A Genome-scale Metabolic model.
2. The starting subsystems or sets of reactions/metabolites defined by the user.
3. Media conditions (aerobic/anaerobic, nitrogen limited, etc.).
4. Possible carbon sources for the studied physiology.
5. Possible by-products or relevant extracellular metabolites. Together with possible carbon sources, these metabolites form a new subsystem that redGEM names as Extracellular Subsystem, this subsystem is treated as other subsystems defined in Step 1 above.
6. Organism specific cofactor pairs.
7. Degree of connection *D* defined by the user.

### redGEM workflow

The central workflow of redGEM involves 4 steps:

1. Choose subsystems (or list of reactions and metabolites, such as synthesis pathway of a target molecule) based on the studied physiology and the part of the metabolism under interest.
2. Derive a new stoichiometric matrix that excludes all cofactor pairs, small metabolites and inorganics.
3. *Identify R^S^*, $R_{ij}^{D},\;R_{ii}^{D}$, *R*^*T*^, and *M*^*S*^, $M_{ij}^{D}$ and $M_{ii}^{D}$ for all subsystem pairs except Extracellular Subsystem.
  ○ Perform a graph search on the new stoichiometric matrix.
  ○ This will find all the links up to degree *D* between each subsystem pairs *S*_*i*_ *and S*_*j*_, and will not find any reaction or metabolites between two subsystems if *L*_*min*,*ij*_ > *D*.
4. To connect all Extracellular Subsystem metabolites to other subsystems, find all reactions $R_{i,j}^{L_{min+n,ij}}$ and all metabolites $M_{\;ij}^{L_{min+n,ij}}$, with *n* as defined by the user.
  ○ If the length of shortest path between a metabolite and *S*_*i*_ is bigger than 1, then:
  ○ ${number\;of\;R}_{ij}^{L_{min+n,ij}}\geq {number\;of\;R}_{i,j}^{n+1}$
  ○ ${number\;of\;of\;M}_{ij}^{L_{min+n,ij}}\geq {number\;of\;M}_{ij}^{n+1}$

The core carbon network is defined as all the reactions and metabolites in *M*^*S*^, $M_{ij}^{D}$ and $M_{ii}^{D}$ (all *i*, *j* pairs), $R^{S^{i}},\;R_{ij}^{D},\;R_{ii}^{D}$ (all *i*, *j* pairs), *R*^*T*^ (reactions that only cofactor pairs, small metabolites and inorganics participate).

### Formulation of biosynthetic lumped reactions for biomass building blocks

We used the lumpGEM algorithm to generate pathways for all biomass building blocks (BBB) as they are defined in GEM. lumpGEM identifies the smallest subnetwork (*S*_*min*_) that are stoichiometrically balanced and capable of synthesizing a biomass building block from defined core metabolites. Moreover, it identifies alternative subnetworks for the synthesis of the same biomass building block. Finally, lumpGEM generates overall lumped reactions, in where the cost of core metabolites, cofactors, small metabolites and inorganics are determined for the biosynthesis. redGEM defined the core network by the algorithm above, and then we generated all minimum sized subnetwork (*S*_*min*_) for each BBB. Then lumpGEM calculated the unique lumped reactions for all the BBBs, and we used these lumped reactions for further validation and other analysis. lumpGEM takes the following steps to build elementally balanced lumped reactions for the biomass building blocks. In the workflow, lumpGEM

1. Decomposes the biomass composition of GEM to each of its components, such as alanine, tyrosine, biotin, etc. In most available GEMs, such decomposition is available mainly in the biomass equation.
2. Builds a new GEM model by allowing the individual production of each *BBB*.
3. Splits all the reactions in GEM in Step a. into forward *F*_*rxn*,*i*_ and backward *B*_*rxn*,*i*_ components.
4. Creates binary variables *z*_*rxn*,*i*_ for each reaction that is defined as non-core by redGEM. Non-core reactions are denoted as *R*^*nC*^.
5. Generates a constraint for each non-core reaction that will control the flux through these reactions as:
  $$F_{rxn,i}+B_{rxn,i}+C∙z_{rxn,i}\leq C$$
  where C is the number of carbon atoms that the cell uptakes from its surrounding. If this quantity is not known, an arbitrary big number can substitute for *C*. When *z*_*rxn*,*i*_ = 1, the reaction is inactive.
6. Applies thermodynamics constraints on the model as defined in[14,16].
7. Builds the following MILP formulation for each BBB:

Maximize

$$\overset{\#\;of\;R^{nC}}{\underset{i}{\sum }}z_{rxn,i}$$

such that:

S.v=0

$$v_{BBB,j}\geq n_{j,GEM}.\mu_{max}$$

where,

*v*_*BBB*,*j*_: The sink that is created in Step 1.a for *BBB*_*j*_ for its biosynthesis.
μ_*max*_: Theoretical maximum specific growth rate for the given physiology in 1/hr units.
*n*_*j*,*GEM*_: The stoichiometric coefficient for *BBB*_*j*_ in mmol/gDW unit as defined in original GEM.

To identify alternative *S*_*min*_ subnetworks for a BBB, lumpGEM further constrains the GEM with the following integer cuts constraint after generating each subnetwork with an iterative manner[59]. The reactions that belong to each subnetwork are denoted as $R_{S_{min}}$

$$\overset{\#\;of\;R_{S_{min}}}{\underset{k}{\sum }}z_{R_{S_{min,k}}}>0$$

### Validation

We validate the consistency between rGEM and GEM performing the following consistency checks by comparing:

1. Theoretical maximum biomass and other by-product of interest yield of rGEM and GEM growing on same carbon source.
  - Under aerobic and anaerobic conditions for the organisms that can grow under both conditions.
2. Essentiality of the common genes between rGEM and GEM.
  - Perform single deletions of the reactions/genes in the rGEM and compare them with GEM.
    - Perform gene essentiality with FBA and with TFA.
3. Allowable flux ranges of the common reactions between rGEM and GEM.
  - Perform Flux Variability Analysis (FVA) and compare the ranges of values of the common reactions between rGEM and GEM.
4. Allowable metabolite and Gibbs free energy of reaction ranges for common metabolite and reactions between rGEM and GEM using TVA.
  - Perform Thermodynamics-based Variability Analysis (TVA) and compare the ranges of substrate/product concentrations and Gibbs free energy of the common reactions between rGEM and GEM.

While these are the basic consistency tests, one could define additional checks, which can be specific to the organism and problem under study. We recommend that in all cases one should perform the checks using FBA and TFA, i.e. with and without thermodynamics constraints.

The first release of the redGEM toolbox is available upon request to the *corresponding author*.

## Supporting information

S1 Fig Concentration variability analysis on rGEM and GEM. The comparison of some common metabolite concentration ranges between rGEM and GEM.
(EPS)

S1 Table D = 1 Core network of *E*. *coli* iJO1366. The core network generated with connection parameter D = 1. This core is the core network used to generate lumped reactions reported in this study.
(XLSX)

S2 Table Lumped reactions generated for D = 1 core network. All the lumped reactions generated for the minimal sized subnetworks for *E*. *coli* iJO1366 with core network of D = 1. There are multiple lumped reactions for many biomass building blocks.
(XLSX)

S1 File The generated D = 1 core model by redGEM. This model do not include the reactions that cannot carry flux under glucose minimal media, moreover it has the transport reactions for the core metabolites across periplasm and extracellular media.
(XLSX)
